# Seed quantity affects the fungal community composition detected using metabarcoding

**DOI:** 10.1038/s41598-022-06997-9

**Published:** 2022-02-23

**Authors:** Funda Oskay, Anna Maria Vettraino, H. Tuğba Doğmuş, Asko Lehtijärvi, Stephen Woodward, Michelle Cleary

**Affiliations:** 1 Faculty of Forestry, Çankırı Karatekin University, 18200 Çankırı, Turkey; 2 Department for Innovation in Biological, Agro-Food and Forest Systems, University of Tuscia, Via S. Camillo de Lellis, Snc, 01100 Viterbo, Italy; 3 Faculty of Forestry, Isparta University of Applied Sciences, 32260 Isparta, Turkey; 4 Isparta University of Applied Sciences, Sütçüler Prof. Dr. Hasan Gürbüz Vocational School, 32950 Isparta, Turkey; 5 School of Biological Sciences, University of Aberdeen, Cruickshank Building, Aberdeen, AB24 3UU Scotland, UK; 6 Southern Swedish Forest Research Centre, Swedish University of Agricultural Sciences, Sundsvägen 3, 230 53 Alnarp, Sweden

**Keywords:** Plant molecular biology, Plant ecology

## Abstract

Pest introductions via trade in tree seed may result from a lack of adequate survey and validation protocols. Developing better diagnostic protocols to identify potentially harmful pests and pathogens in forest tree seed is of critical importance. High-throughput sequencing-based barcoding and metabarcoding provide effective tools for screening potentially harmful organisms in various plant materials, including seeds. However, the sample size needed to detect the total microorganism diversity of a community is a major challenge in microbiome studies. In this work, we examined how increasing sample size (ranging between 100 and 1000 seeds) influences diversity of fungal communities detected by high throughput sequencing in *Pinus sylvestris* seeds. Our results showed that as sample size increased, fungal alpha diversity also increased. Beta-diversity estimators detected significant differences between the mycobiota from different samples. However, taxonomic and functional diversity were not correlated with sample size. In addition, we found that increasing the number of PCR replicates resulted in a higher abundance of plant pathogens. We concluded that for the purpose of screening for potentially harmful pathogens using HTS, greater efforts should be made to increase the sample size and replicates when testing tree seed.

## Introduction

Increased connectivity and globalization have greatly accelerated the frequency and magnitude of biological invasions around the globe^1^. Huge numbers of plants and plant products, many imported from developing countries, circulate within the market, potentially enhancing the spread of alien pests and pathogens^2,3^. For example, plant trade accounts for 57% of pest introductions into Europe alone^3^.

Long-term impacts of invasive alien forest pests and pathogens, including costs for management and derived economic loss, grow inexorably as the number of introductions increases. Phytosanitary requirements for international trade are established by the International Plant Protection Convention (IPPC) through International Standards for Phytosanitary Measures (ISPMs) in order to allow safe plant trade, help prevent the introduction and spread of invasive alien species and advise appropriate measures for control when pests become established. National Plant Protection Organizations (NPPOs) follow the IPPC recommendations and implement ISPMs in administrative and legislative procedures^4^. Based on IPPC recommendations (Agreement on the Application of Sanitary and Phytosanitary Measures), a new European Plant Health Law (Regulation 2016/2031) came into force in December 2019 to ensure safe trade and help mitigate climate change impacts on plant health. The new rules were implemented with several acts, including lists of regulated pests, high-risk plants and plant products and priority pests. Although this new regulation introduces more effective measures than previously in place for the protection of the European territory, discrepancies among member states in the application of the regulations could result in a weakening of the EU biosecurity status^5^. Another issue not considered in the new legislation is the possibility that alien pests and pathogens enter states on either non- or unknown hosts and asymptomatic hosts^6,7^.

As with the previous EU Plant Health Directive (2000/29/EC), EU Regulation 2016/2031 does not provide phytosanitary rules for trade in all forest seeds. Based on the pest risk analysis issued by the European Food and Environment Safety Agency (EFSA), contaminated seeds can be pathways for the potential introduction of quarantine pathogens to disease-free areas. Recently, Cleary, et al.^8^ and Franic, et al.^9^ highlighted that the threat posed by this pathway may be underestimated. Especially for gymnosperms, the production of planting stock is largely through seed, sourced locally or elsewhere, including through e-commerce. Thus, without seed inspection, seed trade could lead to new pest introductions.

Recent DNA-based approaches to identify pests and pathogens in plants and plant products, in particular high-throughput sequencing methods (HTS), have the potential to improve surveys and enable screening for potentially harmful microorganisms in large sample sets^7,10–12^. These methods involve several steps from the field to species detection, with high risks of pitfalls^12,13^. The sample size needed to adequately cover the diversity and community structure of microorganisms is acknowledged as a major challenge in plant microbiome studies^13–15^. Standard protocols for detection of specific pathogens have been developed by international bodies such as the European Plant Protection Organization (EPPO), International Seed Testing Association (ISTA) and the Food and Agriculture Organization (FAO)-IPPC. These protocols can define the sample size needed to detect the organism in question. For example, with *Fusarium circinatum* Nirenberg and O'Donnell, the causal agent of pine pitch canker, EPPO diagnostic protocol PM 7/91 takes into consideration the risk of seed contamination (EPPO Standard PM 7/91)^16^. All steps in the diagnostic process are covered, including that the number of seeds to be tested in a lot should be defined statistically and depends on the specific diagnostic method chosen for pathogen identification. Since counting seeds is demanding, the EPPO protocol suggests that seeds need to be weighed. The mean thousand seed weight suggested, however, ranges from 4 g for *Pinus banksiana* up to 895 g for *P. pinea*. In contrast, international rules for seed testing by ISTA^17^ recommended a minimum of 400 seeds for detecting *F. circinatum* in *Pinus* spp. and *Pseudotsuga menziesii*. For culture dependent detection of this pathogen, sampling strategy using 400 seeds was deemed appropriate to detect an infection of at least 1% with a 95% confidence level^18^. However, it remains unknown what sample volume might be needed to adequately capture the microbial diversity in forest seeds and to inform inspectors about the presence of potentially harmful pathogens when high-throughput sequencing is used.

The aim of this work was to determine if the total fungal community detected in *Pinus sylvestris* varies in samples with different numbers of seeds analysed, hypothesizing that the full coverage is obtained when more seeds are tested.

## Results

### Illumina MiSeq sequencing data

After removal of low-quality reads, short-length, chimeras, plant-derived contaminations and clustering at a 97% similarity level, a total of 1,070,176,680 high-quality reads was obtained. The DNA of sample C was of poor quality and was left out from the analysis. The libraries contained between 1,597,479 and 16,656,791 reads.

### Fungal α-diversity between different amounts of seed samples

**Figure 1:**
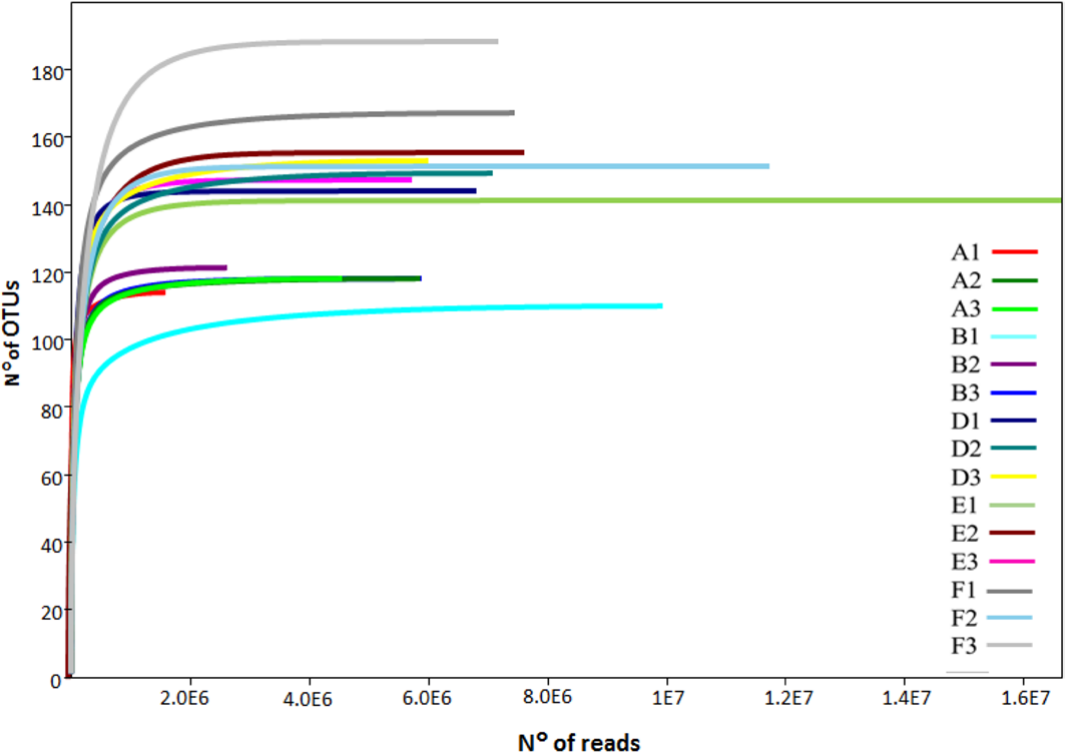
Rarefaction curves of the OTU numbers at 97% similarity for each sample. In the legend, numbers indicate replicates (1, 2, 3) and letters the number of seeds analyzed (A = 100, B = 200; D = 600, E = 800; F = 1000 seeds).

All rarefaction curves approached the saturation plateau, which indicated that the sequencing effort detected the full diversity of the fungal communities (Fig. 1). The correlation between sequencing depth and number of OTUs was not significant (Pearson’s correlation coefficient r = 0.003; P > 0.05). However, rarefaction curves demonstrated that 15 replicates were characterized by a different abundance of OTUs, with the highest values for sample F3.

**Figure 2:**
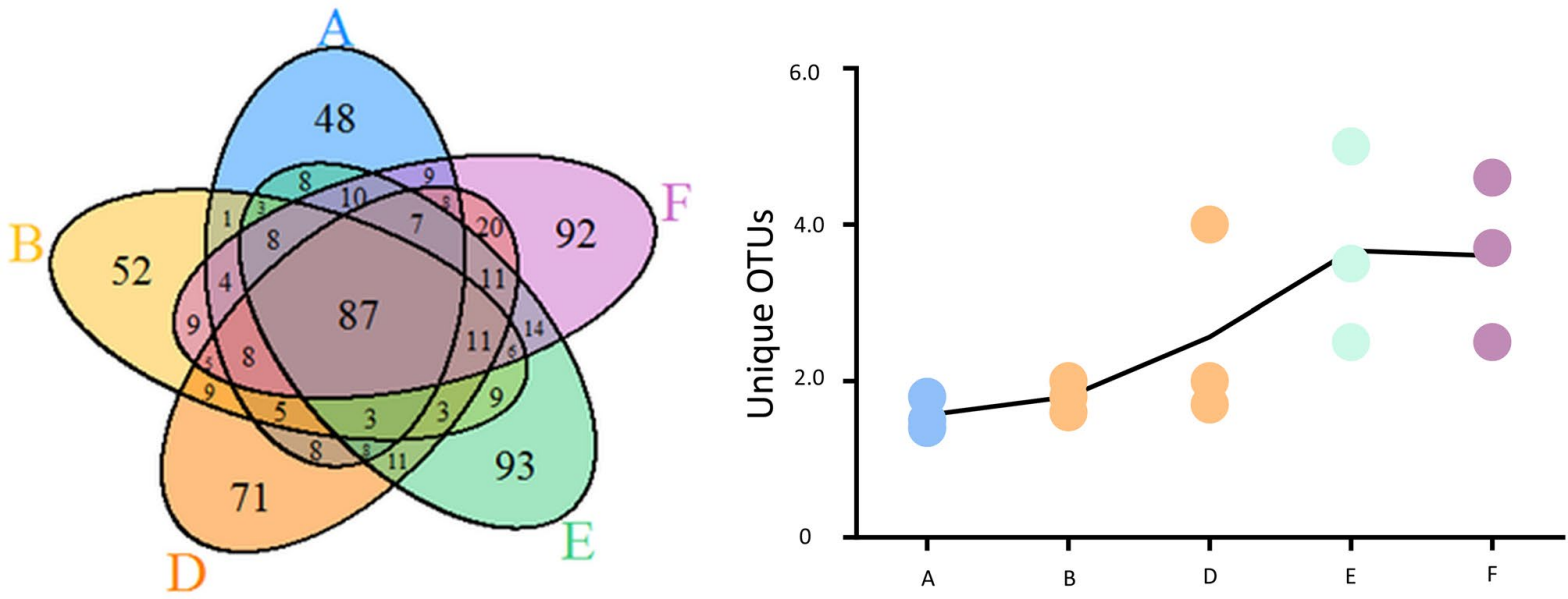
Venn diagram showing the number of shared and unique OTUs among differently sized seed samples (A = 100, B = 200; D = 600, E = 800; F = 1000 seeds) (left) and the unique OTUs abundance for each replicate of different samples (right).

**Figure 3:**
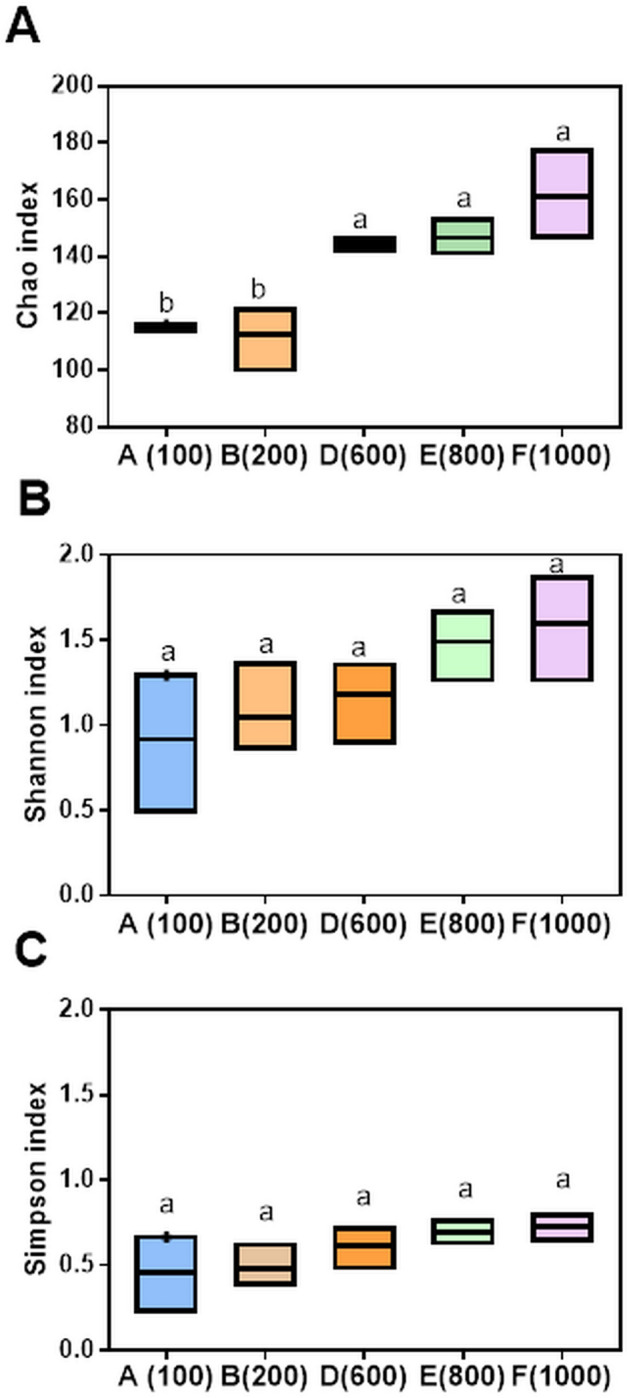
The α-diversity of fungi in Scots pine seed, based on Chao-1 (**A**), Shannon (**B**) and Simpson (**C**) indices. (A = 100, B = 200; D = 600, E = 800; F = 1000 seeds). Differences among five samples were analyzed. The same letters indicate no significant differences (T-test; P < 0.05).

The number of OTUs per sample (A–F) ranged from 101 to 178, with a total of 625 unique OTUs across the dataset. There was a linear correlation between sample size and number of OTUs (Linear regression, R2 = 0.90; P < 0.05). Approximately 14% of the OTUs (87) were shared among all samples (Fig. 2). With increasing sample size, higher numbers of unique OTUs were observed (Pearson’s correlation coefficient r = 0.98, P = 0.002). However, no correlation between the abundance of reads of unique OTUs and sample size was recorded (Pearson’s correlation coefficient r = 0.7; P > 0.05). The number of unique OTUs increased by approximately 70% from sample A and B (Group 1) (approximately 50 unique OTUs/sample) to samples D, E and F (Group 2), (approximately 85 unique OTUs/sample) (Fig. 2). The α-diversity species richness, based on Chao-1, Shannon and Simpson indices, tended to increase as sample size increased (Fig. 3). The pairwise comparison of samples based on the Chao-1 index supported the distinction of samples in two group: Group 1 (samples A and B; extracted from equal to or less than 200 seeds) and Group 2 (samples D, E and F; samples obtained from greater than 600 seeds) (Student t-test, P < 0.05), and were then confirmed by the ANOVA test (post-hoc Tukey test; P < 0.05). Conversely, Shannon and Simpson indices did not differ significantly between samples (Student T-test; P > 0.05) (Fig. 3).

### Fungal diversity among replicates

**Figure 4:**
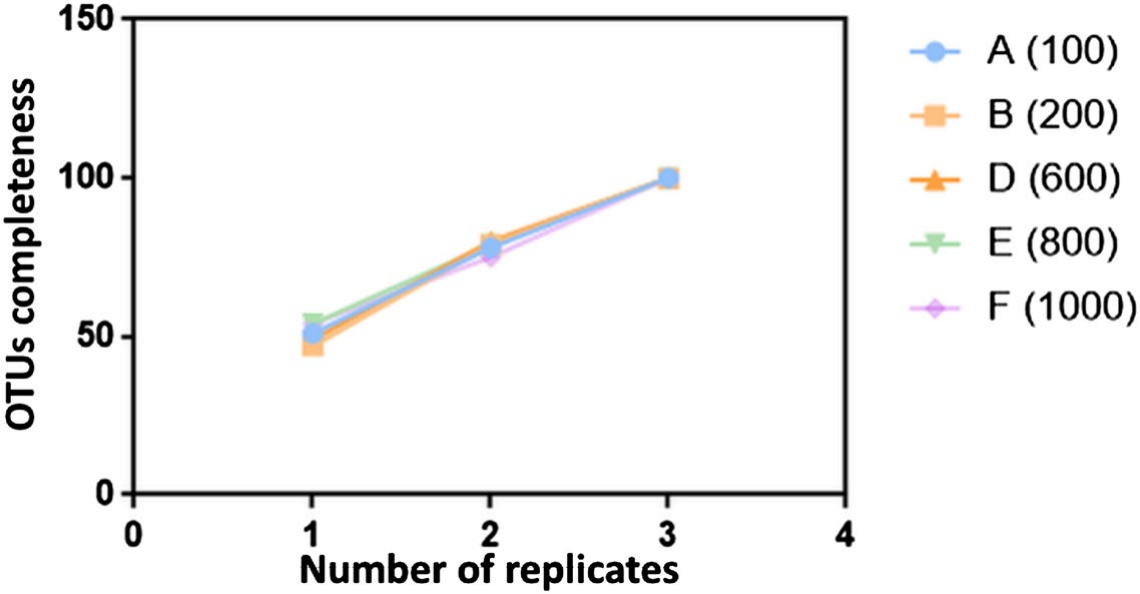
Relative increase in OTUs completeness with each added replication (a total of three) for different amounts of seed samples. [A = 100 seeds (), B = 200 seeds (), D = 600 seeds ( ), E = 800 seeds () and F = 1000 seeds ()].

The average of core OTUs (number of OTUs in all replicates) among three replicates was 20 ± 3%, with no significant differences among samples (Chi-square test, P > 0.05). About 66% (A) to 57% (F) of unique OTUs were only captured in a single sub-sample replicate, and no correlation with sample size was found (Pearson’s correlation coefficient, P** > **0.05). OTUs richness (total number of OTUs) in a sample increased with additional replicates, regardless of the number of seeds sampled. A single replicate captured approximately 50% of the richness. Adding a second replicate and a third replicate led to the apparent richness increasing by approximately 30% and 20%, respectively (Fig. 4). The OTUs present in a single replicate were mainly low-abundant taxa (< 50 reads).

The threshold of proportional abundance required for an OTU to be detected in all 3 PCR replicates was 10% of the total reads of that OTU. The coefficient of variation of α-diversity indices (% CV) among replicates was not associated with sample size (Linear regression; P < 0.05).

### Fungal community structure at OTU level

Differences between communities were evaluated using as explanatory variable the OTUs abundance by the two non-parametric tests of analysis of similarities (ANOSIM) and PERMANOVA. Both analysis confirmed that communities were separated with high dissimilarity between groups (P = 0.0001; 1000 permutations) and separation was large (ANOSIM R = 0.69 and PERMANOVA F-statistics = 3.8).

**Figure 5:**
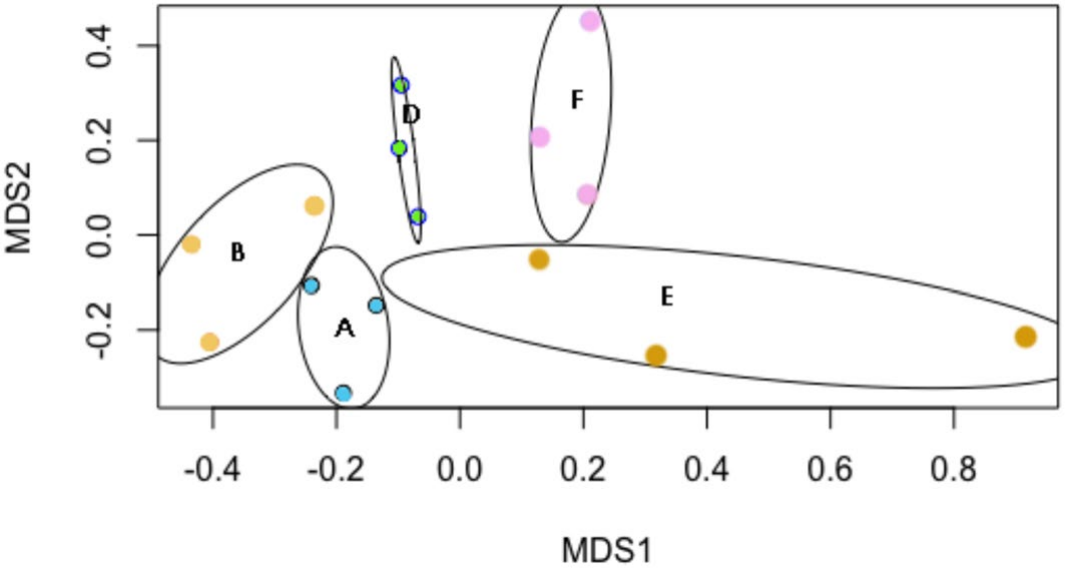
Non-metric multidimensional scaling (NMDS) ordinations based on Bray–Curtis similarities of OTU-based fungal community structures found in *P. sylvestris* seed samples (A = 100, B = 200; D = 600, E = 800; F = 1000 seeds) (Stress value = 0.13).

The potential effects of technical variation on the differentiation of microbial communities at different sample sizes were determined using Non-Metric Multidimensional Scaling analysis (NMDS). Replicates mainly grouped according to the number of seeds (Fig. 5). Samples A and B partially overlapped, whereas samples D, E and F were well separated.

### Fungal community structure at the taxonomic level

OTUs retrieved from seed samples were classified in four phyla: Ascomycota, Basidiomycota, Chytridiomycota and Mucoromycota. Approximately 9% of the OTUs remained unclassified (range: 1–28% among samples). All samples appeared dominated by Ascomycota, which accounted for an average of 90% (range: 88–97% among samples) of the total fungal diversity in the samples, followed by Basidiomycota (range: 0–1% among samples). The relative abundances (read abundance) of Chytridiomycota and Mucoromycota were lower than 0.00001% in all samples. The remaining sequences (9%), consistently detected in all samples, were mostly affiliated to uncultured fungi, matching members of “Fungi spp.”

At the order level, approximately 34% of OTUs were unclassified. Diaporthales and Pleosporales were the most abundant orders with about 14% and 20% of the total reads and they were equally present in all samples (Chi-square test; P > 0.05).

At the genus level, a total of 148 genera were found. The genera *Sydowia* dominated the fungal community (8% of the total OTUs), followed by *Alternaria* (2% of the total OTUs). A total of 32 genera were common to all samples, while 60 genera were detected only on one of the five samples analyzed.

Of the 625 OTUs, 155 were identified at the species level with *Sydowia polyspora* being the most abundant (55% of total reads) followed by *Dothiorella sarmentorum* (15% of total reads), *Epiccoccum nigrum* (6% of total reads) *Alternaria alternata* (4% of total reads) and *Alternaria brassicae* (3% of total reads). Species composition differed between samples, although 14 taxa (*Alternaria alternata*, *Cryptococcus pinus*, C*. tephrensis*, *Dothiorella sarmentorum*, *Epicoccum nigrum*, *Exobasidium maculosum*, *Fusarium avenaceum, Nectria balsamea*, *Pleuroceras pseudoplatani*, *Rosellinia aquila*, *Sarocladium strictum*, *Stagonospora pseudopaludosa*, *Sydowia polyspora*, *Vishniacozyma carnescens*) were common in all samples, with a minimum occupancy in the dataset of approximately 0.0005% of total reads. A minimum portion of the total reads (0.01%) was identified as rare species, i.e. present only in one sample. These reads represent a total of 48% (n = 155) of the taxa identified to species level, and equally distributed among samples (Chi-square test, P > 0.05). Most of the OTUs identified at the species level were considered cosmopolitan species. However, one OTU (matching *Pestalotiopsis citrina)* has been never detected in Europe and was equally distributed in different samples.

### Fungal community structure at the functional level

**Figure 6:**
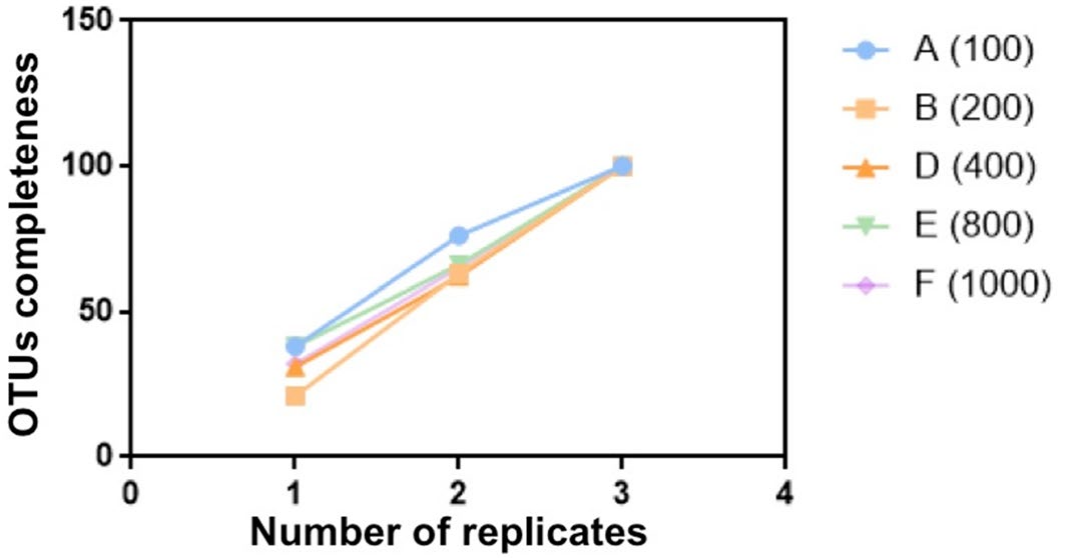
Relative increase in plant pathogens completeness with each added replication (a total of three) for different amounts of seed samples [A = 100 seeds (), B = 200 seeds (), D = 600 seeds (), E = 800 seeds () and F = 1000 seeds ()].

The fungal community colonizing *P. sylvestris* seeds was assessed in terms of fungal guilds and trophic modes. The community was included in six trophic categories with confidence levels including “highly probable” and “probable”, with the category “other” including “possible” and “unclassified” guilds: pathotroph,athotroph-saprotroph, pathotroph-symbiotroph, saprotroph, saprotroph-symbiotroph, symbiotroph. The relative read abundance of saprotroph fungi was higher than that of other groups (mean of the 15 replicates was 56%; range 36–87%). Pathotroph fungi, pathotroph-symbiotroph and pathotroph-saprotroph were less represented (12.4%, 5.2%, 2.6% of the total reads, respectively). There were no significant correlations between fungal functional groups (trophic modes and guilds) and seed samples. Table 1 shows the relative richness of the most abundant functional guilds (> 1% in at least one sample) among samples. The functional guild “Plant Pathogen” included 35 genera out of the 148 recorded genera (24%). However, approximately 27 genera, known to include plant pathogenic species, were classified in other guilds. The genus *Alternaria*, for example, was included in the Animal Pathogen-Endophyte-Plant Pathogen-Wood Saprotroph guild. The abundance (number of OTUs) of plant pathogens in a sample increased with additional replicates, for all samples (Fig. 6). However, the richness in terms of reads assigned to plant pathogens was not significantly affected by sample size (Pearson’s correlation coefficient r = 0.78; P > 0.05).

**Table 1:** Relative richness of the most abundant functional guilds (> 1% in at least one sample) among samples reported as mean (SD).

Functional guild	Samples				
	A	B	D	E	F
Animal Pathogen	0% (0)^b^	0% (0) ^b^	0% (0)^b^	0% (0)^b^	3% (0.016)^a^
Plant Pathogen	15% (0.06)^ac^	5% (0.03)^bc^	8% (0.06)^bc^	24% (0.09)^a^	7% (0.04)^bc^
Undefined Saprotroph	68% (0.21)^ab^	70% (0.09)^a^	48% (0.07)^ab^	53% (0.139)^ab^	39% (0.03)^ab^
> 1 guild	3% (0.01)^a^	7% (0.05)^a^	12% (0.07)^a^	7% (0.08)^a^	7% (0.10)^a^
Other	14% (0.15)^ab^	17% 7 (0.11)^ab^	37% 7(0.03)^a^	16% (0.13)^ab^	38% (0.13)^a^

Different letters after the SD indicate significance at p < 0.05 (Tukey post hoc test).

## Discussion

The high-throughput sequencing methodology used in the current study combined with FUNGuild analyses allowed detection of the DNA of a range of fungal taxa and demonstrated that fungal community diversity depends on the sampling intensity of seeds. This means that current protocols used in phytosanitary screening with a standard sample size (e.g. the EPPO requirement on number of seeds) may not account totally for all possible fungal taxa.

To our knowledge, this is the first work showing that sample size can affect the measurement and interpretation of biodiversity metrics in tree seeds. Safe trade of tree seeds requires adequate surveys and validated protocols in which each step should be well-defined, including the size of samples to be tested. Our results support the findings of previous eDNA studies that the choice of sample size should be considered in detection and monitoring studies. Results showed that samples have significant variability in their fungal microbiotas (beta-diversity) but samples A and B had less variability than samples D through F (NMDS, Chao index). Moreover, larger samples (D-F) accumulated more rare OTUs. Penton, et al.^15^ argued that optimal diversity was detected in large soil samples (10 g soil), capturing rare taxa and decreasing replicate variation, compared with 0.25 and 1 g samples. Similarly, Nascimento, et al.^19^, considering the effects of sample volumes in HTS of aquatic sediments, showed that large samples (> 1% of the sampled area) were required to obtain robust diversity measurement of both metazoan and non-metazoan eukaryotes.

It remains a critical question whether variation among replicates can be fully overcome by increasing sample size in community studies. Several studies focused on the effect of PCR replicates on ecological measures, also with inconsistent results. While many studies using different numbers of PCR replicates reported differences between taxonomic profiles^20–22^, Smith and Peay^23^ concluded that increasing the number of PCR replicates does not affect ecological measures, especially the alpha diversity. However, according to Kauserud et al.^24^, the consistency between replicates might depend on the diversity and complexity of the fungal community considered. The impact of stochasticity during PCR might be higher where a high number of different fungi (and PCR templates) are present.

In our study, PCR replicates increased the possibility to detect new OTUs. In accordance with Shirazi et al.^24^, our study also showed that rare taxa were unique to one or a few replicates of a sample. Moreover, a high value of abundance was not the limit factor for an OTU to be present in all replicates. Thought collectively the unique OTUs account for a small proportion of the total microbiome, we cannot exclude they could play an important role in plant health.

It is worth noticing, that increasing the number of replicates in sampling comes at a cost in terms of finance and time. Nevertheless, replicate numbers should be defined based on sample characteristics. Ficetola, et al.^25^ examining ancient DNA estimated that at least eight PCR replicates should be analysed for samples suspected as having low detection probability. Shirazi et al.^24^ found that even 24 PCR replicates may be insufficient to catalogue biodiversity fully in soil samples and then they recommended a minimum of two PCR replicates to differentiate between sites.

In terms of the taxonomy and functional composition, fungal communities of *P. sylvestris* seeds were similar to those described previously^8^ with the most abundant fungal phylum being Ascomycota. Similarly, a high presence of Diaporthales and Pleosporales was already reported in pine seeds^8^. At the species level, *Sydowia polyspora* dominated fungal communities in seed (relative abundance between 38.5 and 69.5%), which is in agreement with data from previous work on pine seeds^8^.

In this study, we confirm that HTS can be used to detect alien species and plant pathogens. In addition, increasing the sample size will result in a higher number of rare OTUs, and one cannot exclude the possibility that they may be potentially harmful plant pathogens. Thus, while there may be limitations to surveys in terms of resource, in the case of phytosanitary inspections, greater efforts should be made to increase the sample size and replicates when testing tree seed.

We recognize that the practical application of the HTS method, considering the number of samples and replicates that can be processed, will never capture the full diversity in samples, and screening only the DNA (as opposed to RNA) cannot discern the viability of fungal taxa detected. In any case, this method is useful as a rapid and first screening to detect potentially harmful pathogens from which more targeted diagnostics can then be performed (e.g. selective media for culturing, qPCR assays or RNAseq) to confirm identity and viability of pathogens. Albeit we should be aware that those taxa that are ‘unknown’ or yet to be described will inevitably escape our attention.

## Methods

*Pinus sylvestris* seeds, 0.5 kg, collected in 2015 were obtained from a seed orchard in Sweden (Lilla Istad, Öland Island). The pre-determined thousand seed weight (TSW) of the seed lot was 6.2 g, with 100% purity, and a germination rate and viability of 98.50 and 99.75%, respectively. Seeds were not sterilized prior to DNA analyses in order to retain the microbial community on the seed coat which may in itself, serve as an introduction pathway for pathogens.

### DNA extraction, PCR and Illumina amplicon sequencing

Six samples, containing 100, 200, 400, 600, 800 and 1000 seeds (samples A-F), were randomly sorted from the seed lot by weighing the corresponding amount calculated according to the TSW (0.62 to 6.2 g). To allow for small scale heterogeneity within the seed lot, 0.62 g (100 seeds) was taken from the lot at each time and added to the relevant sample until the required final weight was attained (i.e.; 1 × 0.62 g seeds for sample A, 6 × 0.62 g for sample D, 10 × 0.62 g for sample F). Prior to sampling, the 0.5 kg seed lot was transferred to a plastic box, which was surface sterilized using 2% NaOCl (for 10 min) and 70% EtOH (for 5 min), and thoroughly mixed between sub-samplings. Seeds were lyophilized for 48 h in 50 ml Falcon tubes, before homogenizing samples to fine powder in a 25 ml metal grinding container containing a 6 mm diameter stainless steel ball using a mixer mill (Retsch GmbH and Co., KG, Haan, Germany). The metal container and balls were cleaned thoroughly between samples. A single DNA extraction was carried out for each of the six samples, on 30 mg homogenized tissue, using the E.Z.N.A. SP Plant DNA Kit (Omega Bio-tek, Doraville, Georgia) following the manufacturer’s protocol for dry samples. DNA was quantified using a NanoDrop® (ND-2000 UV/vis Spectrophotometer, Thermo Fisher Scientific Inc., Wilmington, Delaware, USA).

The ITS2 region of the rDNA was amplified by PCR using the primers fITS7 (GTGARTCATCGAATCTTTG)^26^ and ITS4 (TCCTCCGCTTATTGATATGC)^27^. For each sample, three replicate PCRs were conducted. Primers were modified by different 5’ identifier sequences of nine base pairs (Macrogen Inc, Seoul, Korea). Reactions were carried out in 50 µl volumes each containing the following reaction components: 5 ng. μl^−1^ template DNA, 200 μM dNTPs; 750 μM MgCl_2_; 10 units Phusion Hot Start polymerase (New England BioLabs, Ipswich, MA, USA), and 200 nM each primer in 1 × buffer. All amplification reactions were performed in an Eppendorf Master Cycler®. The PCR program began with denaturation at 98 °C for 3 min, followed by 31 cycles of 98 °C for 30 s, annealing at 57 °C for 30 s and 72 °C for 30 s, with a final extension step at 72 °C for 7 min. PCR products were purified using Agencourt AMPure XP (Agencourt Bioscience Corp, Massachusetts USA) and quantified using a Qubit 3.0 Fluorometer with the Qubit dsDNA HS Assay Kit (Invitrogen, Carlsbad, CA, USA). After quantification, PCR products were pooled in an equimolar mix and sent for Illumina sequencing, one lane of Miseq 2 × 300 bp (Macrogen Inc, Seoul, Korea).

### Bioinformatics and analysis of sequence data

Sequences were processed using QIIME version 1.7.0^28^. Poor quality reads (< Q20, minimum length = 150 bp, homopolymer = 6 bp) were removed. All sequence files were combined into a single fasta file. Operational taxonomic units [OTUs, sensu^29^] were clustered with 97% similarity cut-off using UPARSE^30^ and chimeric sequences identified and removed in de-novo mode using UCHIME with the default parameters (λ = 2, Minimum score (h) = 0.28; (β) = 8.0; n = 1.4, minimum number of diffs in a segment = 3; minimum divergence = 0.8)^31^. To reduce the effects of sequence number variation, samples were rarefied to the minimum sequencing depth (1 597 479 reads, sample B3) through a subset of randomly selected reads prior to downstream analysis^8^. OTUs were assigned to taxonomic affinities using the Basic Local Alignment Search Tool (BLAST) at the NCBI website (based on > 97% sequence similarity). OTUs were only assigned to species level if the query sequence matched database sequences from fungal isolates (including at least one vouchered specimen) where percentage sequence identity was > 97% and then performed a manual check of the E-values (E^−100^), to ensure no contradictions among different species within the same genus. At lower percentage sequence identity, the query read was identified to genus (identity 95–96%) family (identity 90–94%) order (identity 80–88%) and class level (identity < 80%)^10^. Non fungal-sequences were removed.

### Comparisons of microbial communities and statistical analysis

A rarefaction curve plot was generated showing the number of OTUs versus the number of sequences (reads). The α-diversity of fungal communities was determined using Chao-1, Simpson (D) and the Shannon diversity (H’) indices. Pairwise comparison was done between individual samples using t-test which identified two distinct groups. The values of indices were compared between groups using the ANOVA test with Tukey post hoc analysis (P > 0.05). Linearity of the association of the α-diversity and sample size was determined by linear regression. Correlation between the number of unique OTUs and sample size was assessed by the Pearson’s correlation analysis. Venn diagrams were constructed to visualize the shared fungal communities among all samples. To explore relationships between the fungal community structures and numbers of seeds, PERMANOVA^32^ and ANOSIM^33^ analyses were implemented using a Bray–Curtis distance matrix and the OTUs abundance as explanatory variable. The ANOSIM test statistic (R) ranges from 0 to 1, where the value 0 indicates random grouping between samples and the value 1 indicates 100% dissimilarity between samples.

Fungal community composition among samples and replicates was visualized using Non-Metric Dimensional Scaling (NMDS) on a Bray–Curtis distance matrix.

Several approaches were used to assess the reproducibility of replicates. To estimate OTUs overlap among three replicates the following formula was applied:

$$ {\text{Weighted}}\;{\text{OTU}}\;{\text{overlap}} = \left( {{\text{SOTUS}}_{{1}} + {\text{SOTUS}}_{{2}} + {\text{SOTUS}}_{{3}} } \right)/{\text{TOTUs}} $$

where SOTUS_1_ = the number of sequences within the shared OTUs of replicate 1 shared with other OTUs; SOTUS_2_ = the number of sequences within the shared OTUs of replicate 2 shared with other OTUs; SOTUS_3_ = the number of sequences within the shared OTUs of replicate 3 shared with other OTUs; TOTUs = total number of sequences from replicates 1, 2, and 3.

Dispersion of the variation among replicates was estimated using the coefficient of variation, or ratio of standard deviation to the mean, with the formula CV = s/x̄, where s = standard deviation and x̄ = mean. The higher the value of CV the more dispersion is present. When comparing the OTUs abundance across samples, a Chi-square test was usually used. The identity of OTUs was assessed considering ≥ 97% sequence identify, and ≥ 90% coverage as positive hits.

Each OTU was classified into an ecological guild using FUNGuild^34^ to assess how much variation in community composition was represented by sample size. Those OTUs that were identified with a confidence level designated as “possible”^33^ were included in the category “other” together with the OTUs which remained unclassified. Trophic modes and guilds were described for the OTUs that were identified with a confidence level designated as “highly probable” or “probable”^33^. In addition, OTUs which were placed in more than one guild were included in the “ > 1 guild” category^35^. Finally, relative abundances of microbial taxa and guilds in each sample were calculated and compared using the Chi-square test. The significance level was set at 5%.

The geographical distribution of OTUs identified to a species level was checked using the USDA Fungal Database^36^.

Data were analysed in R version 3.5.1^37^ using vegan package version 2.4^38^, ggplot2^39^ and VennDiagram package^40^.
